# Relationship between Corneal Morphogeometrical Properties and Biomechanical Parameters Derived from Dynamic Bidirectional Air Applanation Measurement Procedure in Keratoconus

**DOI:** 10.3390/diagnostics10090640

**Published:** 2020-08-27

**Authors:** Francisco Cavas, David Piñero, José S. Velázquez, Jorge Mira, Jorge L. Alió

**Affiliations:** 1 Department of Structures, Construction and Graphical Expression, Technical University of Cartagena, 30202 Cartagena, Spain; jose.velazquez@upct.es; 2 Group of Optics and Visual Perception, Department of Optics, Pharmacology and Anatomy, University of Alicante, 03690 Alicante, Spain; david.pinyero@gcloud.ua.es; 3 Doctorate Program in Industrial Technologies, International School of Doctorate, Technical University of Cartagena, 30202 Cartagena, Spain; miraperezjorge@gmail.com; 4 Department of Research and Development, VISSUM, 03016 Alicante, Spain; jlalio@vissum.com; 5 Cornea, Cataract and Refractive Surgery Department, VISSUM, 03016 Alicante, Spain; 6 Division of Ophthalmology, Department of Pathology and Surgery, Faculty of Medicine, Miguel Hernández University, 03202 Alicante, Spain

**Keywords:** keratoconus, corneal hysteresis, corneal biomechanics, corneal volume, corneal morphogeometry

## Abstract

The morphogeometric analysis of the corneal structure has become a clinically relevant diagnostic procedure in keratoconus (KC) as well as the in vivo evaluation of the corneal biomechanical properties. However, the relationship between these two types of metrics is still not well understood. The current study investigated the relationship of corneal morphogeometry and volume with two biomechanical parameters: corneal hysteresis (CH) and corneal resistance factor (CRF), both provided by an Ocular Response Analyzer (Reichert). It included 109 eyes from 109 patients (aged between 18 and 69 years) with a diagnosis of keratoconus (KC) who underwent a complete eye examination including a comprehensive corneal topographic analysis with the Sirius system (CSO). With the topographic information obtained, a morphogeometric and volumetric analysis was performed, defining different variables of clinical use. CH and CRF were found to be correlated with these variables, but this correlation was highly influenced by corneal thickness. This suggests that the mechanical properties of KC cornea contribute only in a partial and limited manner to these biomechanical parameters, being mostly influenced by morphogeometry under normal intraocular pressure levels. This would explain the limitation of CH and CRF as diagnostic tools for the detection of incipient cases of KC.

## 1. Introduction

Morphogeometric analysis of the corneal structure has become a clinically relevant diagnostic procedure in keratoconus, providing a better understanding of the impact that it has on the corneal geometrical data of the degenerative process associated with this disease [1,2,3,4]. This analysis has led to the development of new indices that allow a sensitive and specific detection of keratoconus, even in subclinical stages [1,2,3]. Likewise, this type of analysis has been also used and has demonstrated its usefulness when evaluating and understanding the morphogeometrical changes occurring after some surgical procedures to treat keratoconus [5]. However, the relationship between morphogeometric data and corneal biomechanical properties in keratoconus has not been investigated in depth, one of the reasons being the difficulty in obtaining reliable in-vivo corneal biomechanical data [6]. Two devices are currently being used to obtain measures of corneal biomechanical properties in clinical practice: the Ocular Response Analyzer (Reichert), a dynamic bidirectional applanation device, and the Corvis ST (Oculus), a dynamic Scheimpflug analyzer device [7]. Although these devices have some limitations, especially when trying to determine the real relationship between the measures provided and the standard mechanical properties, they have shown to be useful in detecting and characterizing some changes in keratoconus [8,9,10,11,12,13,14].

Some standard and basic geometric parameters have been found to be correlated with corneal hysteresis (CH) and corneal resistance factor (CRF), two parameters provided by the Ocular Response Analyzer in keratoconus [15,16]. Our research group reported in 2010 a moderate correlation between CRF and mean keratometry in a keratoconus sample (r = −0.564) [16]. A stronger correlation of this biomechanical parameter was found with the level of spherical-like aberrations of the anterior corneal surface in the subgroup of eyes with severe keratoconus (r = −0.655) [16]. Viswanathan et al. [15] reported significant negative correlations of CH (r = −0.43) and CRF (r = −0.53) with anterior maximum keratometry in keratoconic eyes. Likewise, these authors found positive correlations of CH and CRF with central corneal thickness (CCT) and corneal volume (CV) in healthy and keratoconus corneas, confirming that these parameters should be considered as confounding variables [15,17,18,19,20,21,22,23,24]. Indeed, Kamiya et al. [24] demonstrated that the ORA system tends to provide lower CH measurements in eyes with thinner corneas, and consequently a reduced central corneal thickness (CCT) and higher intraocular pressure (IOP). It should be considered that the corneal deformation resulting from applying an air-puff to the cornea (as the ORA system does) has been demonstrated to result from the interaction between the mechanical properties of the corneal structure, IOP, and the morphogeometric profile of such structures [25]. The current study aims to investigate the relationship of different morphogeometric parameters established when considering the cornea as a 3D structure with the biomechanical parameters CH and CRF, and to confirm if this study allows a better understanding of how structural changes induce geometric changes in keratoconus.

## 2. Methods

### 2.1. Measurement Protocol

All patients underwent an extensive ophthalmological examination, which comprised of dilated fundus examination, Goldman’s tonometry, retinoscopy, slit-lamp biomicroscopy, and measure of the CDVA. Corneal hysteresis values were assessed using a G3 model Ocular Response Analyzer (ORA) (Reichert Inc., Buffalo, NY, USA). The procedure of topographical measurement included three consecutive measures made by the same well-experienced technician with a Sirius System^®^ (CSO, Florence, Italy). The only measures that were considered for the study were the ones that showed the best acquisition quality (green-colored checkmarks). Then, the clouds of points representative of both corneal surfaces (anterior and posterior) were saved in colon separated values (.CSV) format, and studied afterward in detail by using a morpho-geometrical analysis procedure established and validated by our research team [1,2,3,4].

### 2.2. Morphogeometric Analysis

**Figure 1:**
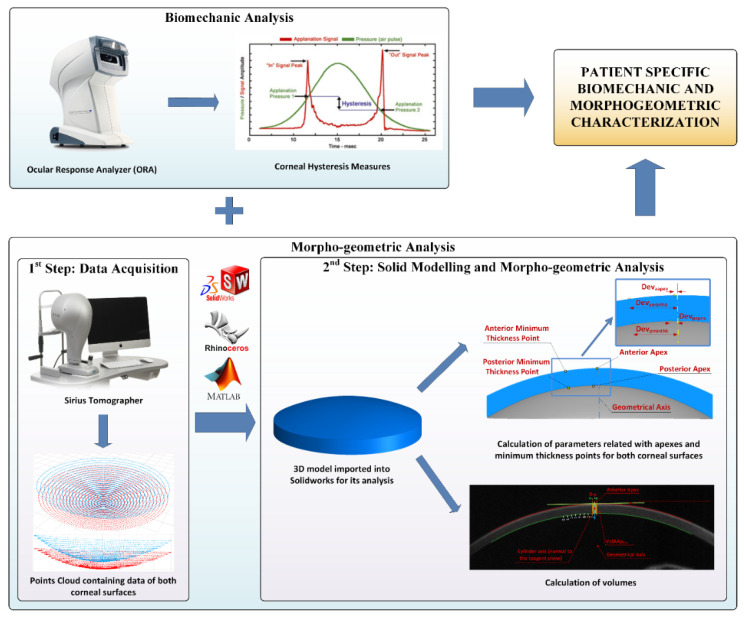
Procedure followed for patient specific biomechanical and morphogeometric characterization. Data obtained from the Ocular Response Analyzer (ORA) and Sirius tomographer allowed us to create a customized 3D model by means of a two-stepped procedure, in which several morphogeometric parameters related with areas and volumes can be studied, along with the corneal hysteresis measures.

The morphogeometric analysis procedure applied had two clearly differenced stages (Figure 1).

#### 2.2.1. Data Acquisition

The tomographer provides data in polar format, so a tailor-made script was programmed in MATLAB^®^ R2018a (Mathworks, Natick, MA, USA) to convert each point of the cloud into Cartesian format. The procedure followed has already been explained in several previous research works [4]. The output obtained is a .CSV file including data of the two clouds of points that represent corneal surfaces for a region comprised between the geometrical center of the cornea (r = 0 mm) and the mid-peripheral area (r = 4 mm). This is the zone in which 97% of abnormalities occur for both healthy and diseased eyes [4]. Then, both clouds of points were exported into the surface reconstruction software Rhinoceros^®^ V 5.0 (MCNeel & Associates, Seattle, WA, USA), and a fitting procedure by the “patch” function (that uses non-uniform rational B-splines, NURBS) was performed to find the surfaces that better approximated the clouds of points.

#### 2.2.2. Solid Modeling and Morpho-Geometric Analysis

In this final stage, a patient-specific 3D model of the cornea was generated using the surfaces previously obtained in Rhinoceros^®^ by exporting them into SolidWorks^®^ V2019 (Dassault Systèmes, Vélizy-Villacoublay, France) software [4]. Once the analysis of the model is done, several morpho-geometrical parameters can be set.

**Figure 2:**
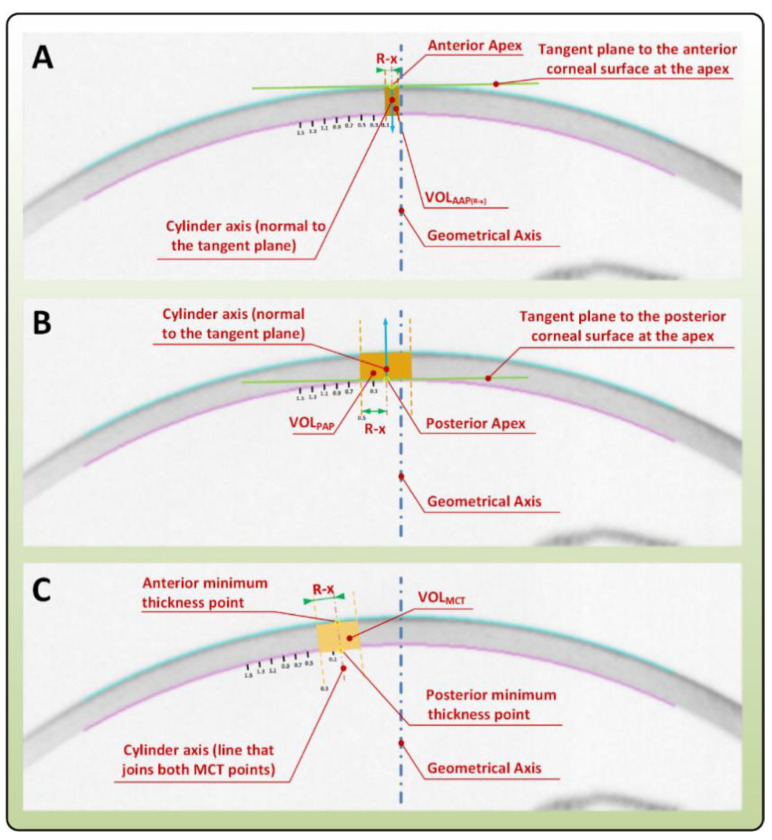
Calculation of VOL_AAP_, VOL_PAP_, and VOL_MCT_ as the intersection between the corneal model and different cylinders of revolution with variable radius, and their axes defined as: (**A**) VOL_AAP_ is a straight line perpendicular to the tangent plane to the anterior corneal surface at the apex (R =  0.1 mm shown); (**B**) VOL_PAP_ is a straight line perpendicular to the tangent plane to the posterior corneal surface at the apex (R  =  0.5 mm shown), (**C**) VOL_MCT_ is the points of the minimum corneal thickness of the anterior and posterior corneal surface (R  =  0.3 mm shown).

The parameters finally used in this study as well as their concept and details have been previously described in several studies of our research group [1,2,3,4]. More precisely, this is the case of the volumetric parameters directly related with volumes around anterior and posterior apices and minimum thickness points used hereafter [3] (Figure 2): A_ant_ is the area of the anterior corneal surface [1,2,4]; A_post_ is the area of the posterior corneal surface [1,2,4]; CV is the total corneal volume [1,2,4]; the sagittal plane apex area (mm^2^) is the area of the cornea within the sagittal plane passing through the Z axis and the highest point (apex) of the anterior (A_apexant_) or posterior (A_apexpost_) corneal surface [2]; the sagittal plane area at minimum thickness point (mm^2^) is the area of the cornea within the sagittal plane passing through the Z axis and the minimum thickness point of the anterior (A_mctant_) and posterior (A_mctpost_) corneal surfaces [2]; the anterior (AAD) and posterior apex deviation (PAD) (mm) are the average distance from the Z axis to the highest point (apex) of the anterior/posterior corneal surfaces [2]; anterior (AMTPD) and posterior minimum thickness point deviation (PMTPD) (mm) are the average distance in the XY plane from the Z axis to the minimum thickness points (maximum curvature) of the anterior/posterior corneal surfaces [1,2,3,4,5]; VOL_MCT_ is the volume contained in the intersection between the solid model of the cornea and a cylinder of revolution with radius × (from 0.1 to 1.5 mm) and its axis is defined by the points of minimum corneal thickness of the anterior and posterior corneal surface [1,2,3,4,5]; VOL_AAP_ is the volume contained in the intersection between the solid model of the cornea and a cylinder of revolution with radius × (from 0.1 to 1.5 mm) and its axis is defined by a straight line perpendicular to the tangent plane to the anterior corneal surface at the apex [1,2,3,4,5]; and VOL_PAP_ is the volume contained in the intersection between the solid model of the cornea and a cylinder of revolution with radius × (from 0.1 to 1.5 mm) and its axis is defined by a straight line perpendicular to the tangent plane to the posterior corneal surface at the apex [1,2,3,4,5].

### 2.3. Patients

This cross-sectional research was made following the directives of the Declaration of Helsinki relative to medical investigation with humans, being approved by the Committee of Ethics of the hospital. It included 109 eyes from 109 patients (ages comprised between 18 and 69) with a diagnosis of keratoconus (KC), all of them being part of the IBERIA database for KC. Data from patients were obtained at the VISSUM clinic in Alicante, Spain, an institution with affiliations with Miguel Hernandez University.

The diagnosis of KC was verified by a highly-experienced ophthalmology professional who looked for the presence of the following evidences: signs of KC in retinoscopy and biomicroscopy (such as Fleischer’s ring, Munson’s sign, Vogt’s striae, Rizzuti’s phenomenon and scissoring), traces of topographical patterns typical of KC on an axial curvature map (irregular, oval, round, superior/inferior-steep without or with skewed radial axes higher than 21 degrees and inferior/superior-steep asymmetric bowtie), focal steepening located around inferior or central/paracentral zones (in both anterior and/or posterior corneal surfaces), and/or significant decrease of corneal thickness reduction, and 3-mm inferior-superior (I-S) mean keratometric difference higher than 1.4 D [26]. Each eye was classified into one of the five possible degrees of severity of the disease, according to the RETICS (Thematic Network for Co-Operative Research in Health) scale, a grading system proposed and validated by Alió et al. [14], which is based mainly in spectacle corrected distance visual acuity (CDVA).

The exclusion criteria included the following conditions and situations: wear of contact lenses during the month previous to the initial visit, signs of dry eye, ocular surface irritation or similar active ocular comorbidity, corneal scarring, corneal thinning disorders, or having undergone any previous ocular surgery.

### 2.4. Statistical Analysis

The software used for statistical analysis purposes was SPSS version 16.0 for Windows (SPSS, Chicago, IL, USA). The Kolmogorov–Smirnov test was selected to assess the normality of the data distributions. One-way analysis of variance (ANOVA) with post-hoc Bonferroni analysis was used to assess the significance of differences between severity in KC subgroups when samples were normally distributed. Otherwise, the Kruskal–Wallis test with a post-hoc analysis performed using the Mann–Whitney test with Bonferroni correction was used. The Pearson or Spearman correlation coefficient was used depending if the normality of data samples could be used or not to analyze the strength of the relationship between different variables in the overall sample. A 2-tailed approach was selected for all statistical tests, and values below 0.05 were required to consider *p*-values to be statistically significant.

Moreover, a multiple regression analysis was performed by means of the backward elimination method with the purpose of finding a mathematical expression that could relate the CH and CRF with morphogeometric and volumetric parameters. Residual analysis was one of the methods selected to evaluate the model’s assumptions, along with the analysis of the normality of unstandardized residuals (homoscedasticity) and the Cook’s distance to detect influential points or outliers. Additionally, the Durbin–Watson test and the estimation of both the collinearity tolerance and the variance inflation factor (VIF) were the methods used to assess the absence of correlation between errors and multicollinearity.

## 3. Results

### 3.1. Descriptive Analysis of the Sample Evaluated

The sample evaluated included a total of 109 KC eyes of 109 patients (mean age: 40.9 years) distributed according to the level of severity of the disease in subgroups as follows: 71 eyes (65.1%) showed signs of early KC (Grade I), 21 (19.3%) with mild KC (Grade II), five (4.6%) with moderate KC (Grade III), and 12 (11.0%) with severe KC (Grade IV). Forty-five patients were females (41.3%) and 64 males (58.7%). The sample included 56 right eyes (51.4%) and 53 left eyes (48.6%). Table 1 shows the characteristics of the evaluated sample according to the level of severity of the disease defined using the RETICS grading system. As shown, statistically significant differences were found in most of the visual, refractive, pachymetric, corneal aberrometric, and topographic data among severity in the KC subgroups (*p* ≤ 0.025). Likewise, significant differences were found between severity in KC subgroups in CH and CRF (*p* < 0.001). Specifically, significant differences were found in CRF for the comparisons of Grades I–III (*p* = 0.012), Grades I–IV (*p* < 0.001), and Grades II–IV (*p* = 0.012). Concerning CH, only significant differences were found between Grades I and IV (*p* < 0.001). No statistically significant differences were found between the KC severity subgroups in terms of IOP (*p* = 0.162).

### 3.2. Morphogeometric Analysis

Table 2 shows a summary of the morphogeometrical data obtained in the evaluated sample according to the level of severity of the disease defined using the RETICS grading system. As shown, statistically significant differences among the KC subgroups were found in most of the morphogeometric parameters (*p* ≤ 0.048). Only A_apexant_ (*p* = 0.055) and PAD (*p* = 0.085) did not differ significantly among severity in the KC subgroups. When comparisons were performed between pairs of KC subgroups, significant differences between Grades I and II were detected for A_ant_ (*p* = 0.012), A_post_ (*p* = 0.036), and AAD (*p* = 0.006). When comparing the Grade I and III KC subgroups, significant differences were observed in A_ant_ (*p* < 0.001), A_post_ (*p* < 0.001), A_apexpost_ (*p* = 0.012), A_mctant_ (*p* = 0.018), A_mctpost_ (*p* = 0.012), AMTPD (*p* = 0.018), and PMTPD (*p* = 0.036). Furthermore, significant differences between the Grades I and IV KC subgroups were found in A_ant_ (*p* < 0.001), A_post_ (*p* < 0.001), AAD (*p* < 0.001), AMTPD (*p* = 0.024), and PMTPD (*p* = 0.036). Likewise, significant differences were additionally found for the following comparisons: Grades II–III (A_ant_, *p* = 0.03), Grades II–IV (A_ant_, *p* < 0.001, A_post_, *p* < 0.001), and Grades III–IV (corneal volume, *p* = 0.049).

Table 3 shows a summary of the volumetric data obtained in the evaluated sample according to the level of severity of the disease defined using the RETICS grading system. As shown, statistically significant differences among the KC subgroups were found in all volumetric parameters (*p* < 0.001). Specifically, all VOL_MCT_, VOL_AAP_, and VOL_PAP_ differed significantly between Grades I and III (*p* < 0.001), Grades I and IV (*p* < 0.001), and Grades II and IV (*p* ≤ 0.030) in the KC subgroups. Furthermore, VOL_AAP_ and VOL_PAP_ for radii of 0.2, 0.3, 0.4, 0.5, and 0.6 mm differed significantly among the Grade II and III KC subgroups (*p* ≤ 0.048). Statistically significant differences between the Grades II and III KC subgroups were found in VOL_AAP_ for a radius of 0.1 mm (*p* = 0.048) as well as in VOL_PAP_ for radii of 0.7 (*p* = 0.030) and 0.8 mm (*p* = 0.036).

### 3.3. Correlation between Morphogeometric and ORA Biomechanical Data

A statistically significant correlation of CH with CCT (r = 0.685, *p* < 0.001) and minimal corneal thickness (MCT) (r = 0.665, *p* < 0.001) was found. Likewise, CRF was significantly correlated with CCT (r = 0.785, *p* < 0.001) and MCT (r = 0.770, *p* < 0.001). Concerning volumetric data, all VOL_MCT_, VOL_AAP_, and VOL_PAP_ values correlated significantly with CH (r ≥ 0.589, *p* < 0.001) and CRF (r ≥ 0.703, *p* < 0.001). However, these correlations were poor when controlled for CCT and MCT (−0.173 ≤ r ≤ 0.121, *p* ≥ 0.095).

CH was also found to be significantly correlated with CV (r = 0.463, *p* < 0.001), A_ant_ (r = −0.381, *p* < 0.001), A_post_ (r = −0.356, *p* < 0.001), A_apexpost_ (r = 0.588, *p* < 0.001), A_mctant_ (r = 0.595, *p* < 0.001), A_mctpost_ (r = 0.594, *p* < 0.001), and AAD (r = −0.258, *p* = 0.007). Similarly, CRF was significantly correlated with the same parameters: CV (r = 0.437, *p* < 0.001), A_ant_ (r = −0.459, *p* < 0.001), A_post_ (r = −0.467, *p* < 0.001), A_apexpost_ (r = 0.608, *p* < 0.001), A_mctant_ (r = 0.609, *p* < 0.001), A_mctpost_ (r = 0.609, *p* < 0.001) and AAD (r = −0.345, *p* < 0.001). As with the volumetric data, these correlations became poor when controlled for CCT and MCT (−0.049 ≤ r ≤ 0.206, *p* ≥ 0.063).

### 3.4. Multiple Linear Regression Analysis

CH was found to be significantly related to different morphogeometric and volumetric data obtained according to the following expression (*p* < 0.001):

CH = −2.37 − 0.60 × CV + 6.41 × A_mctant_ (Adjusted R^2^:0.399) (1)

The homoscedasticity of this model was confirmed by the normality of the unstandardized residual distribution (*p* = 0.200) and the absence of influential points or outliers (mean Cook’s distance = 0.009 ± 0.021). Residual values were below 1 and 1.5 mm Hg in 50.5% and 76.1% of cases, respectively.

Likewise, CRF was found to be significantly related to different morphogeometric and volumetric data following this mathematical expression (*p* < 0.001):

CRF = −6.21 − 0.72×CV − 0.12×A_apexant_ + 7.71×A_mctant_ + 0.92×PMTPD (Adjusted R^2^:0.519) (2)

The homoscedasticity of this model was confirmed by the normality of the unstandardized residual distribution (*p* = 0.200) and the absence of influential points or outliers (mean Cook’s distance = 0.011 ± 0.023). Residual values were below 1 and 1.5 mm Hg in 54.1% and 82.6% of cases, respectively.

## 4. Discussion

CH and CRF have been used for many years as parameters indirectly characterizing the mechanical properties of the cornea [6,7]. However, some limitations in their diagnostic ability for detecting some corneal pathologies such as keratoconus [27] have been reported, especially in mild or subclinical cases [28]. Corneal thickness and volume have been identified as confounding variables for CH and CRF, with the potential of CRF being useful for KC detection once the effect of corneal thickness on this parameter is considered [29]. Likewise, the results of finite element analyses showed that both CH and CRF were significantly correlated with corneal elastic modulus, relaxation limit, and relaxation time, confirming the viability of a mechanical interpretation of these parameters [30]. The aim of the current study was to characterize the relationship between the ORA biomechanical parameters that indirectly represent the mechanical properties of the cornea, and the morphogeometric and volumetric parameters developed by our research group [1,2,3,4]. These parameters, derived from a comprehensive morphogeometric analysis of the cornea, have been shown to be an accurate approach for clinical and subclinical keratoconus diagnosis [2,3].

In the sample evaluated, although most of the morphogeometric parameters showed significant differences between the KC severity subgroups like in previous studies [2,4], A_ant_ statistically showed significant differences in almost all comparisons between pairs of subgroups. This suggests that this parameter may have better capacity to discriminate between different levels of severity of the disease when compared to the other evaluated parameters. This may make sense, considering that most of the changes occurring in the cornea as the disease progresses and becomes more severe take place on the anterior corneal surface [14,26]. Likewise, VOL_AAP_ and VOL_PAP_ for radii of 0.2, 0.3, 0.4, 0.5, and 0.6 mm for the cylinder of revolution used for the calculation of volume also showed statistically significant differences in most of the comparisons between pairs of KC severity subgroups. This is consistent with the results of previous studies conducted by our research group, in which we followed the same volumetric approach in other KC populations, showing a further reduction of corneal volume in keratoconus that significantly progressed along the disease severity level [3].

Moderate to strong correlations of ORA biomechanical parameters with pachymetric and volumetric data were found, which is consistent with the results of previous studies [15,19,23]. Viswanathan et al. [15] demonstrated in a prospective comparative study that CH and CRF were influenced by the corneal structure, with higher values in corneas with greater thickness and volume. Likewise, Rosa and colleagues [19] suggested that, according to the outcomes of clinical research on healthy subjects, CH and CRF were related to the corneal shape and thickness, showing CH decreased with age. Considering that thickness decreased as the severity of the keratoconus increased, the capacity of CRF to detect significant differences between some KC severity subgroups seems coherent. However, it should be considered that corneal deformation resulting from applying an air-puff to the cornea (as the ORA system does) is the consequence of the interaction between the mechanical properties of the corneal structure, IOP, and the morphogeometric profile of such a structure [25]. This type of testing does not seem to be sufficient to accurately define the individual contribution of each factor [25].

Significant correlations were found between most of the morphogeometric parameters evaluated and CH and CRF. This confirms the relevance of the morphogeometric and volumetric profile of the cornea in the measurement of CH and CRF in keratoconus, and more specifically, the relevance of the corneal thickness profile. Indeed, all these correlations became poor and statistically non-significant when the correlation analysis was performed, but controlling for MCT and CCT. Furthermore, multiple linear regression analysis confirmed that CH and CRF could be predicted in keratoconus eyes with acceptable accuracy from some morphogeometric and volumetric parameters, confirming the partial contribution of the mechanical properties of the cornea to these ORA parameters. Better prediction (R2: 0.519) was obtained for CRF, with a linear relationship dependent on corneal volume, A_apexant_, A_mctant_, and PMTPD. More studies are needed to validate these predictive models in other kerotoconic samples as well as to define if these trends are also observed in other corneal pathological conditions.

In conclusion, the ORA biomechanical parameters CH and CRF are correlated with morphogeometric and volumetric parameters in keratoconus corneas, but this correlation is highly influenced by corneal thickness. This suggests that there is only a partial and limited contribution of the mechanical properties of the keratoconus cornea to these parameters, being mostly influenced by morphogeometry, considering normal IOP levels. This would explain the limitation of CH and CRF as diagnostic parameters of keratoconus in the most incipient cases in which the pachymetric reduction is still non-existent or very limited.

## Figures and Tables

**Table 1:** Summary of the characteristics of the evaluated sample according to the level of severity of the disease defined using the RETICS (Thematic Network for Co-Operative Research in Health) grading system. Abbreviations: UDVA, uncorrected distance visual acuity; SE, spherical equivalent; CDVA, corrected distance visual acuity; IOP, intraocular pressure; CH, corneal hysteresis; CRF, corneal resistance factor; RMS, root mean square; HOA, high order aberrations; SA, spherical aberration; Q, asphericity; MCT, minimum corneal thickness; CCT, central corneal thickness; D, diopters.

Mean (SD)	Grade I	Grade II	Grade III	Grade IV	*p*-Value
Median (Range)					
Age (years)	41.4 (19.2)	41.4 (21.4)	55.4 (31.5)	30.8 (9.0)	0.372
	36.0 (14 to 98)	33.0 (15 to 82)	51.0 (23 to 94)	33.0 (15 to 43)	
LogMAR UDVA	0.56 (0.52)	1.00 (0.64)	1.12 (0.62)	1.48 (0.77)	<0.001
	0.39 (−0.18 to 2.00)	0.91 (0.24 to 2.00)	1.00 (0.28 to 2.00)	1.50 (0.40 to 3.00)	
Sphere (D)	−0.95 (2.34)	−1.96 (3.34)	−1.06 (2.35)	−5.13 (7.20)	0.327
	−0.50 (−8.00 to 5.00)	−0.75 (−9.50 to 3.00)	−0.25 (−4.50 to 0.75)	−3.00 (−20.00 to 3.00)	
Cylinder (D)	−2.28 (2.02)	−3.23 (2.15)	−4.63 (1.70)	−4.88 (4.95)	0.025
	−2.00 (−9.00 to 0.00)	−2.88 (−8.25 to 0.00)	−4.25 (−7.00 to −3.00)	−3.25 (−17.00 to 0.00)	
SE (D)	−2.09 (2.45)	−3.58 (3.29)	−3.38 (2.28)	−7.56 (7.34)	0.019
	−1.75 (−9.75 to 4.00)	−2.88 (−11.25 to 1.50)	−2.38 (−6.75 to −2.00)	−4.75 (−21.75 to 0.00)	
LogMAR CDVA	0.09 (0.17)	0.29 (0.30)	0.51 (0.26)	0.72 (0.37)	<0.001
	0.00 (−0.18 to 0.77)	0.21 (−0.04 to 1.00)	0.51 (0.18 to 0.82)	0.59 (0.36 to 1.30)	
IOP (mm Hg)	12.15 (2.15)	11.42 (1.98)	10.80 (2.50)	11.25 (2.34)	0.162
	12.00 (8 to 17)	11.00 (9 to 15)	10.00 (9 to 15)	10.50 (8 to 16)	
CH (mm Hg)	8.75 (1.56)	8.25 (1.57)	7.40 (0.93)	6.73 (0.88)	<0.001
	8.60 (5.20 to 12.20)	8.50 (5.50 to 11.50)	7.50 (6.50 to 8.80)	6.45 (5.70 to 8.70)	
CRF (mm Hg)	7.68 (1.61)	6.91 (1.54)	5.30 (1.09)	5.13 (1.12)	<0.001
	7.70 (3.80 to 12.20)	7.20 (3.20 to 9.70)	5.30 (4.20 to 6.90)	5.20 (3.40 to 7.40)	
Corneal RMS HOA (µm)	1.84 (1.09)	2.97 (1.58)	4.42 (1.37)	6.47 (2.51)	<0.001
	1.72 (0.42 to 5.07)	3.36 (0.35 to 5.72)	3.56 (3.24 to 6.05)	5.35 (3.38 to 9.88)	
Corneal coma RMS (µm)	1.46 (1.07)	2.48 (1.56)	3.59 (1.61)	5.04 (2.44)	<0.001
	1.39 (0.05 to 4.97)	2.48 (0.21 to 5.58)	2.57 (2.26 to 5.51)	4.03 (1.99 to 9.49)	
Corneal SA (µm)	0.17 (0.34)	−0.25 (0.70)	−0.93 (0.94)	−2.35 (1.60)	<0.001
	0.24 (−1.38 to 1.03)	−0.04 (−1.60 to 0.69)	−1.46 (−1.70 to 0.44)	−2.20 (−5.88 to 0.49)	
Anterior Q 4.5 mm	−0.02 (1.42)	−0.86 (1.53)	−1.90 (1.54)	−1.85 (1.12)	<0.001
	−0.02 (−4.40 to 4.10)	−0.85 (−4.52 to 2.35)	−2.00 (−4.25 to −0.20)	−1.90 (−3.15 to 0.68)	
Anterior Q 8 mm	−0.48 (0.53)	−1.01 (0.86)	−1.67 (0.66)	−2.26 (0.32)	<0.001
	−0.44 (−2.20 to 0.62)	−1.00 (−2.42 to 0.28)	−1.72 (−2.68 to −0.93)	−2.35 (−2.80 to −1.74)	
MCT (µm)	469.4 (46.3)	438.5 (50.2)	366.2 (40.1)	344.2 (49.6)	<0.001
	471.0 (316 to 570)	436.5 (363 to 529)	375.0 (307 to 416)	337.5 (264 to 444)	
CCT (µm)	487.4 (44.2)	458.8 (51.7)	386.4 (43.0)	372.3 (41.5)	<0.001
	485.0 (335 to 588)	459.0 (371 to 532)	385.0 (321 to 432)	367.5 (300 to 447)	

**Table 2:** Summary of the morphogeometric data obtained in the evaluated sample according to the level of severity of the disease defined using the RETICS grading system. Abbreviations: SD, standard deviation; A_ant_, anterior corneal surface area; A_post_, posterior corneal surface area; CV, corneal volume; A_apexant_, sagittal plane area at anterior apex; A_apexpost_, sagittal plane area at posterior apex; A_mctant_, sagittal plane area at anterior minimum thickness point; A_mctpost_, sagittal plane area at posterior minimum thickness point; AAD: anterior apex deviation; PAD, posterior apex deviation; AMTPD, anterior minimum thickness point deviation; PMTPD, posterior minimum thickness point deviation.

Mean (SD)	Grade I	Grade II	Grade III	Grade IV	*p*-Value
Median (Range)					
A_ant_ (mm^2^)	43.25 (0.21)	43.49 (0.27)	44.04 (0.35)	44.57 (0.54)	<0.001
	43.26 (42.66 to 43.62)	43.48 (43.08 to 44.02)	44.24 (43.52 to 44.35)	44.50 (43.81 to 45.51)	
A_post_ (mm^2^)	44.54 (0.34)	44.85 (0.46)	45.69 (0.53)	46.55 (0.81)	<0.001
	44.58 (43.85 to 45.32)	44.91 (44.21 to 45.84)	45.74 (44.87 to 46.36)	46.38 (45.13 to 47.85)	
CV (mm^3^)	23.73 (1.64)	23.32 (1.65)	21.65 (2.65)	24.22 (1.47)	0.048
	23.63 (19.13 to 27.62)	23.24 (19.09 to 27.60)	22.60 (16.97 to 23.27)	24.11 (21.86 to 27.98)	
A_apexant_ (mm^2^)	2.09 (1.98)	3.29 (1.19)	2.66 (1.53)	3.83 (0.27)	0.055
	3.19 (0.00 to 4.69)	3.63 (0.00 to 4.49)	3.00 (0.00 to 3.82)	3.84 (3.21 to 4.25)	
A_apexpost_ (mm^2^)	3.94 (0.29)	3.85 (0.28)	3.54 (0.32)	3.80 (0.24)	0.013
	3.90 (3.18 to 4.66)	3.87 (3.19 to 4.48)	3.68 (3.00 to 3.83)	3.73 (3.31 to 4.15)	
A_mctant_ (mm^2^)	3.93 (0.29)	3.84 (0.29)	3.53 (0.33)	3.78 (0.27)	0.015
	3.89 (3.14 to 4.64)	3.73 (3.17 to 4.48)	3.67 (2.99 to 3.83)	3.73 (3.19 to 4.15)	
A_mctpost_ (mm^2^)	3.93 (0.29)	3.84 (0.29)	3.53 (0.33)	3.78 (0.27)	0.014
	3.90 (3.13 to 4.65)	3.73 (3.17 to 4.48)	3.67 (2.99 to 3.82)	3.73 (3.19 to 4.15)	
AAD (mm)	0.008 (0.013)	0.017 (0.016)	0.019 (0.019)	0.027 (0.022)	<0.001
	0.002 (0.000 to 0.060)	0.011 (0.000 to 0.060)	0.014 (0.000 to 0.040)	0.020 (0.000 to 0.070)	
PAD (mm)	0.17 (0.09)	0.19 (0.10)	0.18 (0.09)	0.24 (0.09)	0.085
	0.16 (0.04 to 0.46)	0.18 (0.03 to 0.38)	0.21 (0.05 to 0.29)	0.28 (0.08 to 0.40)	
AMTPD (mm)	1.06 (0.37)	0.93 (0.40)	0.59 (0.28)	0.74 (0.23)	0.002
	1.04 (0.34 to 2.20)	0.83 (0.31 to 1.83)	0.55 (0.23 to 1.00)	0.78 (0.38 to 1.14)	
PMTPD (mm)	0.99 (0.35)	0.86 (0.38)	0.54 (0.29)	0.68 (0.23)	0.003
	0.97 (0.32 to 2.08)	0.78 (0.28 to 1.72)	0.52 (0.20 to 0.97)	0.71 (0.30 to 1.07)	

**Table 3:** Summary of the volumetric data obtained in the evaluated sample according to the level of severity of the disease defined using the RETICS grading system. Abbreviations: SD, standard deviation; VOL_MCT_, anterior corneal surface area; corneal volume defined by the points of minimal thickness; VOL_AAP_, corneal volume defined by the anterior corneal apex; VOL_PAP_, corneal volume defined by the posterior corneal apex. These volumes were calculated for different radius values of the revolution cylinder, ranging from 0.1 to 1.5 mm.

Mean (SD)	Grade I	Grade II	Grade III	Grade IV	*p*-Value
Median (Range)					
**VOL_MCT_ (mm^3^)**					
Radius 0.1 mm	0.015 (0.001)	0.014 (0.002)	0.011 (0.002)	0.011 (0.002)	<0.001
	0.015 (0.010 to 0.020)	0.014 (0.011 to 0.020)	0.012 (0.009 to 0.013)	0.011 (0.008 to 0.014)	
0.2	0.058 (0.005)	0.055 (0.006)	0.046 (0.005)	0.044 (0.007)	<0.001
	0.059 (0.042 to 0.070)	0.055 (0.045 to 0.070)	0.047 (0.038 to 0.050)	0.044 (0.032 to 0.060)	
0.3	0.13 (0.01)	0.12 (0.01)	0.10 (0.01)	0.10 (0.01)	<0.001
	0.13 (0.10 to 0.16)	0.12 (0.11 to 0.15)	0.11 (0.09 to 0.12)	0.10 (0.07 to 0.13)	
0.4	0.23 (0.02)	0.22 (0.03)	0.19 (0.02)	0.18 (0.03)	<0.001
	0.24 (0.17 to 0.29)	0.22 (0.18 to 0.27)	0.19 (0.15 to 0.21)	0.18 (0.13 to 0.22)	
0.5	0.37 (0.03)	0.35 (0.04)	0.29 (0.03)	0.28 (0.04)	<0.001
	0.37 (0.27 to 0.45)	0.35 (0.29 to 0.42)	0.30 (0.24 to 0.33)	0.28 (0.21 to 0.35)	
0.6	0.53 (0.05)	0.50 (0.06)	0.42 (0.05)	0.41 (0.06)	<0.001
	0.53 (0.39 to 0.65)	0.50 (0.42 to 0.60)	0.43 (0.35 to 0.47)	0.41 (0.31 to 0.51)	
0.7	0.72 (0.07)	0.69 (0.08)	0.58 (0.06)	0.56 (0.07)	<0.001
	0.73 (0.52 to 0.88)	0.69 (0.57 to 0.82)	0.59 (0.48 to 0.65)	0.56 (0.44 to 0.70)	
0.8	0.95 (0.09)	0.90 (0.10)	0.76 (0.09)	0.75 (0.10)	<0.001
	0.95 (0.69 to 1.16)	0.90 (0.75 to 1.07)	0.78 (0.63 to 0.85)	0.74 (0.58 to 0.92)	
0.9	1.21 (0.11)	1.15 (0.13)	0.97 (0.11)	0.96 (0.12)	<0.001
	1.21 (0.88 to 1.47)	1.15 (0.96 to 1.36)	1.00 (0.80 to 1.09)	0.95 (0.76 to 1.17)	
1	1.50 (0.13)	1.41 (0.18)	1.21 (0.14)	1.21 (0.14)	<0.001
	1.50 (1.10 to 1.82)	1.43 (1.00 to 1.68)	1.24 (1.00 to 1.36)	1.20 (0.97 to 1.46)	
1.1	1.82 (0.16)	1.76 (0.19)	1.48 (0.17)	1.48 (0.17)	<0.001
	1.82 (1.34 to 2.21)	1.75 (1.45 to 2.04)	1.52 (1.21 to 1.66)	1.47 (1.21 to 1.78)	
1.2	2.18 (0.19)	2.09 (0.21)	1.78 (0.20)	1.80 (0.20)	<0.001
	2.17 (1.61 to 2.64)	2.09 (1.74 to 2.44)	1.83 (1.46 to 1.99)	1.78 (1.49 to 2.14)	
1.3	2.58 (0.22)	2.46 (0.26)	2.11 (0.24)	2.15 (0.23)	<0.001
	2.57 (1.91 to 3.11)	2.48 (2.00 to 2.87)	2.17 (1.72 to 2.36)	2.13 (1.80 to 2.54)	
1.4	3.01 (0.25)	2.90 (0.28)	2.48 (0.29)	2.54 (0.26)	<0.001
	2.99 (2.23 to 3.62)	2.91 (2.40 to 3.36)	2.54 (2.02 to 2.76)	2.51 (2.14 to 2.97)	
1.5	3.47 (0.28)	3.36 (0.32)	2.88 (0.34)	2.97 (0.28)	<0.001
	3.46 (2.59 to 4.18)	3.36 (2.77 to 3.90)	2.95 (2.33 to 3.21)	2.94 (2.52 to 3.44)	
**VOL_AAP_ (mm^3^)**					
Radius 0.1 mm	0.015 (0.001)	0.014 (0.002)	0.012 (0.001)	0.012 (0.001)	
	0.015 (0.011 to 0.018)	0.014 (0.012 to 0.017)	0.012 (0.010 to 0.014)	0.012 (0.009 to 0.014)	<0.001
0.2	0.061 (0.005)	0.057 (0.006)	0.048 (0.005)	0.047 (0.005)	
	0.061 (0.040 to 0.070)	0.058 (0.050 to 0.070)	0.047 (0.040 to 0.060)	0.047 (0.040 to 0.060)	<0.001
0.3	0.14 (0.01)	0.13 (0.01)	0.11 (0.01)	0.11 (0.01)	
	0.14 (0.10 to 0.17)	0.13 (0.11 to 0.15)	0.11 (0.09 to 0.12)	0.11 (0.08 to 0.13)	<0.001
0.4	0.24 (0.02)	0.23 (0.02)	0.19 (0.02)	0.19 (0.02)	
	0.24 (0.17 to 0.30)	0.23 (0.19 to 0.27)	0.20 (0.16 to 0.22)	0.19 (0.15 to 0.23)	<0.001
0.5	0.38 (0.03)	0.36 (0.04)	0.30 (0.03)	0.30 (0.03)	
	0.38 (0.27 to 0.46)	0.36 (0.30 to 0.42)	0.31 (0.25 to 0.34)	0.30 (0.24 to 0.35)	<0.001
0.6	0.55 (0.05)	0.52 (0.05)	0.44 (0.05)	0.44 (0.05)	
	0.55 (0.39 to 0.67)	0.52 (0.43 to 0.60)	0.44 (0.36 to 0.50)	0.43 (0.35 to 0.51)	<0.001
0.7	0.75 (0.06)	0.71 (0.07)	0.60 (0.07)	0.60 (0.06)	
	0.75 (0.54 to 0.91)	0.71 (0.59 to 0.82)	0.60 (0.49 to 0.68)	0.59 (0.49 to 0.70)	<0.001
0.8	0.98 (0.08)	0.93 (0.10)	0.79 (0.09)	0.79 (0.08)	
	0.98 (0.71 to 1.19)	0.94 (0.78 to 1.07)	0.79 (0.65 to 0.89)	0.78 (0.65 to 0.92)	<0.001
0.9	1.24 (0.10)	1.18 (0.12)	1.00 (0.11)	1.01 (0.10)	
	1.24 (0.90 to 1.50)	1.19 (0.99 to 1.36)	1.01 (0.82 to 1.13)	1.00 (0.84 to 1.18)	<0.001
1	1.54 (0.12)	1.47 (0.15)	1.24 (0.14)	1.27 (0.12)	
	1.53 (1.12 to 1.86)	1.48 (1.23 to 1.68)	1.26 (1.02 to 1.41)	1.25 (1.05 to 1.46)	<0.001
1.1	1.86 (0.15)	1.78 (0.18)	1.52 (0.17)	1.55 (0.14)	
	1.86 (1.37 to 2.26)	1.80 (1.51 to 2.04)	1.53 (1.24 to 1.71)	1.54 (1.30 to 1.79)	<0.001
1.2	2.23 (0.18)	2.13 (0.21)	1.82 (0.21)	1.88 (0.16)	
	2.22 (1.64 to 2.69)	2.15 (1.81 to 2.45)	1.84 (1.49 to 2.05)	1.86 (1.58 to 2.15)	<0.001
1.3	2.62 (0.21)	2.52 (0.24)	2.16 (0.25)	2.23 (0.18)	
	2.62 (1.94 to 3.16)	2.54 (2.14 to 2.90)	2.18 (1.76 to 2.42)	2.21 (1.90 to 2.55)	<0.001
1.4	3.05 (0.24)	2.93 (0.28)	2.53 (0.29)	2.63 (0.20)	
	3.04 (2.28 to 3.67)	2.97 (2.51 to 3.40)	2.55 (2.05 to 2.83)	2.60 (2.24 to 2.98)	<0.001
1.5	3.52 (0.27)	3.39 (0.31)	2.93 (0.34)	3.06 (0.23)	
	3.51 (2.65 to 4.22)	3.43 (2.89 to 3.94)	2.95 (2.37 to 3.28)	3.03 (2.64 to 3.46)	<0.001
**VOL_PAP_ (mm^3^)**					
Radius 0.1 mm	0.015 (0.001)	0.014 (0.002)	0.012 (0.001)	0.011 (0.002)	<0.001
	0.015 (0.011 to 0.018)	0.014 (0.011 to 0.017)	0.012 (0.010 to 0.013)	0.012 (0.009 to 0.014)	
0.2	0.060 (0.005)	0.057 (0.006)	0.047 (0.005)	0.045 (0.006)	<0.001
	0.060 (0.040 to 0.070)	0.057 (0.050 to 0.070)	0.047 (0.040 to 0.050)	0.047 (0.040 to 0.060)	
0.3	0.14 (0.01)	0.13 (0.01)	0.11 (0.01)	0.10 (0.01)	<0.001
	0.14 (0.10 to 0.16)	0.13 (0.10 to 0.15)	0.11 (0.09 to 0.12)	0.10 (0.08 to 0.13)	
0.4	0.24 (0.02)	0.23 (0.03)	0.19 (0.02)	0.18 (0.02)	<0.001
	0.24 (0.17 to 0.29)	0.23 (0.19 to 0.27)	0.19 (0.16 to 0.21)	0.18 (0.15 to 0.22)	
0.5	0.38 (0.03)	0.36 (0.04)	0.30 (0.03)	0.29 (0.04)	<0.001
	0.38 (0.27 to 0.46)	0.36 (0.29 to 0.42)	0.30 (0.25 to 0.34)	0.29 (0.23 to 0.35)	
0.6	0.54 (0.05)	0.52 (0.06)	0.43 (0.05)	0.42 (0.05)	<0.001
	0.54 (0.39 to 0.66)	0.52 (0.42 to 0.61)	0.43 (0.36 to 0.48)	0.42 (0.34 to 0.51)	
0.7	0.74 (0.06)	0.71 (0.08)	0.59 (0.06)	0.58 (0.07)	<0.001
	0.74 (0.53 to 0.90)	0.71 (0.58 to 0.83)	0.59 (0.49 to 0.66)	0.57 (0.47 to 0.70)	
0.8	0.97 (0.08)	0.93 (0.10)	0.77 (0.09)	0.77 (0.09)	<0.001
	0.97 (0.70 to 1.18)	0.93 (0.77 to 1.09)	0.78 (0.64 to 0.87)	0.76 (0.63 to 0.92)	
0.9	1.23 (0.10)	1.18 (0.13)	0.99 (0.11)	0.98 (0.11)	<0.001
	1.23 (0.89 to 1.49)	1.19 (0.98 to 1.38)	1.00 (0.81 to 1.11)	0.97 (0.82 to 1.18)	
1	1.52 (0.12)	1.45 (0.18)	1.23 (0.14)	1.23 (0.13)	<0.001
	1.52 (1.11 to 1.85)	1.47 (1.00 to 1.70)	1.24 (1.01 to 1.38)	1.22 (1.03 to 1.46)	
1.1	1.85 (0.15)	1.80 (0.19)	1.50 (0.17)	1.51 (0.15)	<0.001
	1.85 (1.36 to 2.24)	1.80 (1.50 to 2.07)	1.51 (1.23 to 1.68)	1.49 (1.28 to 1.78)	
1.2	2.21 (0.18)	2.14 (0.22)	1.80 (0.20)	1.83 (0.17)	<0.001
	2.21 (1.62 to 2.67)	2.15 (1.80 to 2.47)	1.82 (1.47 to 2.02)	1.81 (1.56 to 2.14)	
1.3	2.60 (0.21)	2.50 (0.28)	2.13 (0.24)	2.18 (0.20)	<0.001
	2.60 (1.93 to 3.14)	2.54 (2.00 to 2.91)	2.16 (1.74 to 2.39)	2.15 (1.87 to 2.54)	
1.4	3.03 (0.24)	2.95 (0.29)	2.50 (0.29)	2.58 (0.22)	<0.001
	3.02 (2.27 to 3.66)	2.97 (2.49 to 3.38)	2.53 (2.03 to 2.79)	2.54 (2.23 to 2.98)	
1.5	3.50 (0.28)	3.39 (0.34)	2.90 (0.34)	3.01 (0.25)	<0.001
	3.49 (2.60 to 4.21)	3.42 (2.87 to 3.92)	2.93 (2.35 to 3.24)	2.97 (2.62 to 3.45)	
